# Supplementary material for: Artistoo, a library to build, share, and explore simulations of cells and tissues in the web browser
Source: eLife. 2021 Apr 9;10:e61288. doi: 10.7554/eLife.61288 (PMC8143789; doi:10.7554/eLife.61288)
Supplement: Transparent reporting form [file elife-61288-transrepform.docx]

***eLife’s* transparent reporting form**

We encourage authors to provide detailed information *within their submission* to facilitate the interpretation and replication of experiments. Authors can upload supporting documentation to indicate the use of appropriate reporting guidelines for health-related research (see [EQUATOR Network](http://www.equator-network.org/%20)), life science research (see the [BioSharing Information Resource](https://biosharing.org/)), or the [ARRIVE guidelines](http://www.plosbiology.org/article/info:doi/10.1371/journal.pbio.1000412) for reporting work involving animal research. Where applicable, authors should refer to any relevant reporting standards documents in this form.

If you have any questions, please consult our Journal Policies and/or contact us: [editorial@elifesciences.org](mailto:editorial@elifesciences.org).

**Sample-size estimation**

- You should state whether an appropriate sample size was computed when the study was being designed
- You should state the statistical method of sample size computation and any required assumptions
- If no explicit power analysis was used, you should describe how you decided what sample (replicate) size (number) to use

Please outline where this information can be found within the submission (e.g., sections or figure legends), or explain why this information doesn’t apply to your submission:

We have not conducted a power analysis because we do not perform any statistical testing or inference in this manuscript. We only provide descriptive summary statistics (mean, standard deviation) to give readers an indication of the time needed to run simulations. Since this wall time is relatively consistent for simulations of the same size and parameters, we chose an (arbitrary) number of 5 simulations to compute these summary statistics from.

**Replicates**

- You should report how often each experiment was performed
- You should include a definition of biological versus technical replication
- The data obtained should be provided and sufficient information should be provided to indicate the number of independent biological and/or technical replicates
- If you encountered any outliers, you should describe how these were handled
- Criteria for exclusion/inclusion of data should be clearly stated
- High-throughput sequence data should be uploaded before submission, with a private link for reviewers provided (these are available from both GEO and ArrayExpress)

Please outline where this information can be found within the submission (e.g., sections or figure legends), or explain why this information doesn’t apply to your submission:

Each simulation was performed in 5 independent runs as stated in the figure legend. The distinction between biological and technical replicates does not apply to simulations; when fixing the parameter settings (fully described in the Supplementary Methods and the supplied model files), independent runs only differ in their random seed. Model files are provided in the supplementary data.

We have no outliers, excluded data, or sequencing data to report.

**Statistical reporting**

- Statistical analysis methods should be described and justified
- Raw data should be presented in figures whenever informative to do so (typically when N per group is less than 10)
- For each experiment, you should identify the statistical tests used, exact values of N, definitions of center, methods of multiple test correction, and dispersion and precision measures (e.g., mean, median, SD, SEM, confidence intervals; and, for the major substantive results, a measure of effect size (e.g., Pearson's r, Cohen's d)
- Report exact p-values wherever possible alongside the summary statistics and 95% confidence intervals. These should be reported for all key questions and not only when the p-value is less than 0.05.

Please outline where this information can be found within the submission (e.g., sections or figure legends), or explain why this information doesn’t apply to your submission:

The manuscript contains no statistical analyses beyond the computation of means and standard deviations. All statistical information required to interpret the figures (definition of center and dispersion, N) can be found in the figure legend. We compute no p-values in this manuscript because it is not our aim to perform statistical inference; we only report summary statistics to give readers an estimate of simulation run times.

(For large datasets, or papers with a very large number of statistical tests, you may upload a single table file with tests, Ns, etc., with reference to sections in the manuscript.)

**Group allocation**

- Indicate how samples were allocated into experimental groups (in the case of clinical studies, please specify allocation to treatment method); if randomization was used, please also state if restricted randomization was applied
- Indicate if masking was used during group allocation, data collection and/or data analysis

Please outline where this information can be found within the submission (e.g., sections or figure legends), or explain why this information doesn’t apply to your submission:

Group allocation is not applicable when “samples” are simulations, where all “experimental groups” (i.e., the framework or grid size) are perfectly controllable and there is no possibility of confounding requiring randomization.

**Additional data files (“source data”)**

- We encourage you to upload relevant additional data files, such as numerical data that are represented as a graph in a figure, or as a summary table
- Where provided, these should be in the most useful format, and they can be uploaded as “Source data” files linked to a main figure or table
- Include model definition files including the full list of parameters used
- Include code used for data analysis (e.g., R, MatLab)
- Avoid stating that data files are “available upon request”

Please indicate the figures or tables for which source data files have been provided:

Source data files are provided for figure 2C-D, allowing users to reproduce the simulations in both Morpheus and Artistoo. For morpheus simulations, we used the xml model files that are shipped with the (free) Morpheus software, and for Artistoo stimulations we provide node scripts in our online Github repository: <https://github.com/ingewortel/artistoo-supplements>. Analysis code is not provided since we do not perform any analyses beyond computing means and standard deviations.
